# Complete Genome Sequences of Four *Brucella* Strains Isolated from China

**DOI:** 10.1128/genomeA.01034-17

**Published:** 2017-10-12

**Authors:** Xiaowen Yang, Xiaofang Cao, Ning Wang, Jiawei Wang, Pengfei Bie, Yanli Lyu, Qingmin Wu

**Affiliations:** a Key Laboratory of Animal Epidemiology and Zoonosis of the Ministry of Agriculture, College of Veterinary Medicine, China Agricultural University, Beijing, China; b Animal Science and Technology College, Beijing University of Agriculture, Beijing, China

## Abstract

*Brucella* spp. are facultative intracellular pathogens that cause a contagious zoonotic disease. Twelve different species are currently identified. This study presents the complete genome sequences of four *Brucella* strains. These complete genomes were annotated and the contents compared to those of other strains isolated from China.

## GENOME ANNOUNCEMENT

*Brucella* spp., facultative intracellular pathogens that can persistently colonize animal host cells, cause zoonosis that can result in outcomes such as abortion or sterility in susceptible animal hosts (1). Twelve different species are currently identified, *B. melitensis*, *B. abortus*, *B. suis*, *B. ovis*, *B. canis*, *B. ceti* (2), *B. pinnipedialis* (3), *B. neotomae* (4), *B. microti* (5), *B. inopinata* (4), *B. papionis* (6), and *B. vulpis* (7). Until now, there were nine strains isolated from China. In this study, we performed whole-genome sequencing of two *B. melitensis* strains and two *B. abortus* strains isolated from China.

The whole genomes of these strains were sequenced using the Illumina sequencing platform using a 250-bp paired-end library, with at least 100-fold (100×) coverage to obtain the raw data. Raw data were used with FastQC to test the sequencing quality, and redundant and low-quality sequences were removed. SAMtools (8) was used to output the depth and coverage of sequencing. By comparing the sequencing depth and coverage, the closed genomes were identified (9). The paired-end reads were assembled *de novo* using the Velvet (10) software. The contigs were marked by NCBI BLAST to confirm the sites in the closed genomes. Meanwhile, the gaps were amplified using primers designed by Vector NTI (Invitrogen). These data were submitted to the NCBI Prokaryotic Genome Annotation Pipeline (PGAP) for annotation (11).

*B. melitensis* BY38 was isolated from sheep in Bayingolin Mongol Autonomous Prefecture, Xinjiang Uygur Autonomous Region, and identified in 2013. The complete genome had two chromosomes, and the total genome was 3,311,862 bp, the average GC content was 57.2%, the coverage was 99.99%, and the sequencing depth was 181.65×. A total of 3,305 coding sequences (CDSs), 9 rRNAs, 55 tRNAs, and 4 noncoding RNAs (ncRNAs) were annotated. The most closely related genome was that of *B. melitensis* M28.

*B. melitensis* BL was isolated from a dairy cow in Altay Prefecture, Xinjiang Uygur Autonomous Region, and identified in 2012. The complete genome also had two chromosomes, and the total genome was 3,312,673 bp, the average GC content was 57.2%, the coverage was 99.95%, and the sequencing depth was 195.91×. A total of 3,310 CDSs, 9 rRNAs, 55 tRNAs, and 4 ncRNAs were annotated. The most closely related genome was that of *B. melitensis* 63/9.

*B. abortus* BD was isolated from a dairy cow in Baoding city, Hebei Province, and identified in 2008. The total genome was 3,271,067 bp, the average GC content was 57.2%, the coverage was 99.84%, and the sequencing depth was 158.28×. A total of 3,271 CDSs, 9 rRNAs, 55 tRNAs, and 4 ncRNAs were annotated. The most closely related genome was that of *B. abortus* BAB8416.

*B. abortus* MC was isolated from a dairy cow in Mancheng city, Hebei Province. The total genome was 3,312,673 bp, which was shorter than that of *B. abortus* BD. The average GC content of genome was 57.2%, the coverage was 99.79%, and the sequencing depth was 157.65×. A total of 3,267 CDSs, 9 rRNAs, 55 tRNAs, and 4 ncRNAs were annotated. The most closely related genome was also that of *B. abortus* BAB8416.

### Accession number(s).

The complete genome sequences were deposited in NCBI GenBank under accession numbers CP022827 and CP022828, CP022875 and CP022876, CP022877 and CP022878, and CP022879 and CP022880 for *B. melitensis* BY38, *B. melitensis* BL, *B. abortus* BD, and *B. abortus* MC, respectively.
